# Mitochondrial dysfunction, alternans, and arrhythmias

**DOI:** 10.3389/fphys.2013.00083

**Published:** 2013-04-19

**Authors:** Miguel A. Aon

**Affiliations:** School of Medicine, Johns Hopkins UniversityBaltimore, MD, USA

The second law [of thermodynamics] is one of the all-time great laws of science, for it illuminates why anything—anything from the cooling of hot matter to the formulation of a thought—happens at all.

Peter Atkins.

Four laws that drive the universe

It seems already like a long time ago that we used to think about heart failure as a problem in which mitochondria and energy were not involved. Energy has been for so long granted in the mind of mainstream electrophysiologists, but not anymore. Although energy can be taken for granted under normal conditions, this is not the case in heart failure where dysfunctional mitochondria, lack of substrate, and downstream effects become dominant factors. In the last decade, this unrealistic assumption has dramatically changed. Increasing experimental evidence supports the role of mitochondrial malfunction in the origin of arrhythmias (Aon et al., 2003; Akar et al., 2005; O'Rourke et al., 2005; Jeong et al., 2012; Aggarwal and Makielski, 2013).

A push forward of this idea came in 2010, when *Frontiers in Physiology* published the work of Florea and Blatter (2010), which showed that the propensity for pacing-induced Ca^2+^ alternans in atrial myocytes increases when the mitochondrial membrane potential (ΔΨ_m_) is dissipated or ATP synthesis inhibited. Alternans—a well-established risk factor for ventricular and atrial dysrhythmias—occur in cardiac failure and during myocardial ischemia. Hindering either of the two main components of the energy transducing machinery of mitochondria: the energy flow (ATP) and its main driving force (ΔΨ_m_), trigger *ailleurs* Ca^2+^ alternans, or the beat-to-beat variations in Ca^2+^ transient amplitude at constant stimulation frequency. Further enhancement of alternans also occurred after inhibition of respiratory complexes or the Ca^2+^-sensitive dehydrogenases from the tricarboxylic acid cycle. Together, these data suggested that factors diminishing mitochondrial Ca^2+^ sequestration generate favorable conditions for Ca^2+^ alternans. Affecting mitochondrial Ca^2+^ handling by decreasing either uptake through the uniporter or extrusion through the Na^+^/Ca^2+^ exchanger also increased alternans. Then, Florea and Blatter made the following intriguing observation: alternans may happen in the absence of effects on the sarcoplasmic reticulum (SR) Ca^2+^ replenishment, and despite impaired mitochondrial energetics and Ca^2+^ dynamics. They suggested that under these conditions cytosolic ATP levels are still sufficiently high to allow for normal sarcoendoplasmic reticulum Ca^2+^ ATPase (SERCA) activity and/or ATP for SERCA is supplied by glycolysis.

In more recent work, Florea and Blatter (2012) investigated pacing-induced alternans to further explore the role exerted by ATP supply from glycolysis or oxidative phosphorylation or both, under β-adrenergic stimulation in cat atrial myocytes. They show that beta stimulation abolishes Ca^2+^ alternans even in the presence of selective inhibition of either glycolytic or mitochondrial ATP supply, both enhancers of alternans occurrence. Only severe deficit of energy supply given by simultaneous inhibition of both catabolic pathways prevented β-adrenergic stimulation-mediated abrogation of Ca^2+^ alternans. The authors suggest that enhanced Ca^2+^ sequestration together with β-adrenergic-mediated effects on the ryanodine receptor appear to act in tandem to protect against pacing-induced Ca^2+^ alternans. In fact, all cellular and transport processes involving subcellular organelles that contribute to clear cytosolic Ca^2+^ protect against alternans, and that conditions that impair cytosolic Ca^2+^ sequestration promote alternans. Impairment of mitochondrial Ca^2+^ uptake during excitation-contraction (EC) coupling can potentially have an impact on alternans incidence, although its quantitative importance is still a matter of debate. In this scenario, beta adrenergic receptor stimulation triggers a tug of war between protective and enhancing effects on alternans, the former through increase in Ca^2+^ sequestration and the latter by augmenting SR load and fractional release.

Mitochondrial redox and energetic functions are inextricably linked, and both are liable in heart dysfunction in chronic diseases such as obesity and diabetes (Bugger and Abel, 2010; Tocchetti et al., 2012). It is now well established that significant perturbations in the mitochondrial redox environment trigger mitochondrial ΔΨ_m_ depolarization that under critical conditions can scale up to the whole heart, thereby producing fatal arrhythmias (Aon et al., 2009; Kembro et al., in press). Reactive oxygen species (ROS) affect cardiac ion channels, cytoplasmic ionic balance, contractile proteins, and EC coupling (Christians and Benjamin, 2012; Aggarwal and Makielski, 2013). In this context, the work of Florea and Blatter poses several key questions worth investigating. What is the redox-dependence of the pacing-induced alternans? Since ROS also reduces the ability of SERCA and plasma membrane Ca^2+^ ATPase to sequester cytosolic Ca^2+^ back into the SR or its efflux from the cell (Aggarwal and Makielski, 2013), could the propensity of cells to exhibiting alternans increase under oxidative challenge or pathological conditions such as cardiac hypertrophy? Is mitochondrial ROS signaling involved in the alternans occurrence? These questions point to the pivotal role of mitochondria in health, disease, aging, and their potential for generating, under critical conditions, a higher propensity to arrhythmias.
